# Telemedicine and Mortality Reduction During COVID-19: Telemonitoring as a Key Strategy for Emergency Health care Preparedness

**DOI:** 10.1177/26924366251374495

**Published:** 2025-08-28

**Authors:** Andrea Foppiani, Valeria Calcaterra, Simona Bertoli, Alberto Battezzati, Marco Frontini, Gianvincenzo Zuccotti

**Affiliations:** ^1^ International Center for the Assessment of Nutritional Status and the Development of Dietary Intervention Strategies (ICANS-DIS), Department of Food, Environmental and Nutritional Sciences (DeFENS), University of Milan, Milan, Italy.; ^2^ IRCCS Istituto Auxologico Italiano, Clinical Nutrition Unit, Department of Endocrine and Metabolic Medicine, Milan, Italy.; ^3^ Pediatric and Adolescent Unit, Department of Internal Medicine, University of Pavia, Pavia, Italy.; ^4^ Pediatric Department, Buzzi Children’s Hospital, Milan, Italy.; ^5^ IRCCS Istituto Auxologico Italiano, Obesity Unit and Laboratory of Nutrition and Obesity Research, Department of Endocrine and Metabolic Diseases, Milan, Italy.; ^6^ Link Up s.r.l., Milano, Italy.; ^7^ Department of Biomedical and Clinical Science, University of Milan, Milan, Italy.

**Keywords:** COVID-1, health care, mortality, telemedicine, telemonitoring

## Abstract

**Introduction:**

Telemedicine, particularly remote monitoring, offers a promising approach to enhance health care. This study evaluated the impact of the Operations Center for Discharged Patients (COD19) telemonitoring service on COVID-19 patient mortality during the pandemic, exploring telemedicine’s potential in managing severe health emergencies.

**Patients and Methods:**

A retrospective analysis was conducted on COVID-19 patients in home isolation, divided into telemonitoring and non-telemonitoring groups. All-cause mortality was the primary outcome. The COD19 provided active surveillance for the telemonitoring group.

**Results:**

The study included 6,017 patients: 2,431 telemonitored and 3,586 non-telemonitored. Telemonitored patients were older and had more comorbidities, including cardiovascular and metabolic diseases, and a higher risk of hospitalization. Critically, mortality was significantly lower in the telemonitored group (1.3% vs. 2.9%, *p* < 0.001). Multivariable analysis confirmed telemonitoring’s significant reduction of death risk, while age, sex, and comorbidities increased it.

**Conclusions:**

Proactive at-home telemonitoring correlates with reduced mortality in COVID-19 patients. The COVID-19 pandemic highlighted telemedicine’s potential as a vital strategy for emergency health care readiness.

## Introduction

The COVID-19 pandemic, a major disruption of the decade, brought unforeseen global socioeconomic impacts.^1^ It placed an unprecedented strain on Italy’s health care system, particularly in Lombardy, the epicenter of the outbreak.^2,3^ The region faced a rapid surge in infections, leading to a critical shortage of hospital beds and intensive care units. The situation was further complicated by the closure of outpatient clinics, limiting access to routine health care services. The pandemic’s impact extended beyond the health care system, causing widespread concern and isolation among the population.^4^ The need for effective home-based care solutions became paramount as hospitals struggled to cope with the influx of patients.

The Operations Center for Discharged Patients (COD19) and COD20 systems were developed in response to this crisis, aiming to bridge the gap between hospital and community care.^5^ These systems enabled remote monitoring of patients’ clinical conditions, provided access to telemedicine services, and facilitated communication between patients and health care providers. By leveraging technology, COD19 and COD20 helped to alleviate the burden on hospitals, ensured that patients received timely and appropriate care, and supported the management of the pandemic in Italy.

Telemedicine and remote patient monitoring (RPM) extend beyond the mere transmission of health data through a remote connection; they constitute an innovative care model that enhances the quality of healthcare delivery and strengthens the effectiveness of clinical interventions.^6,7^

RPM is a strategy of telemedicine that offers a way for clinicians to observe patients’ physiological parameters remotely and to intervene if abnormalities appear.^6^ Telemonitoring applications are valuable for continuous patient monitoring between clinic visits, enabling early identification of deterioration and prompt care.^8–11^ This is especially beneficial for chronic conditions, improving outcomes and encouraging preventive habits.^12,13^ RMP also reduces unnecessary clinic visits and enhances practitioner efficiency by providing real-time patient data.^14^ RPM technology allows clinicians to access crucial symptom and condition information that patients might not report during in-person visits. It provides a more accurate health assessment by mitigating issues like “white coat syndrome,” leading to better treatment decisions.^15^ In addition, long-term data storage helps track health trends and personalize care.

However, most of the existing literature focuses on stable, non-emergency scenarios and on long-term chronic conditions.^16–19^ Few studies at the time had evaluated the real-time use of RPM as a public health response tool for managing infectious disease outbreaks.^20–24^ Moreover, most previous work did not address the integration of telemonitoring into public health systems, and only a few studies have analyzed its effectiveness in reducing mortality in high-pressure, resource-constrained settings.^8,25^

Our study addresses this gap by providing evidence of how a structured telemonitoring service (COD19) can contribute to reducing mortality during the COVID-19 pandemic in Milan (Lombardy, Italy), offering insights into how telemedicine can be strategically implemented in future emergency response models.

## Materials and Methods

### Study design

This was a retrospective cohort study examining the impact of a telemonitoring service on COVID-19 mortality during the second wave of the SARS-CoV-2 pandemic in Milan, Italy. Patients diagnosed with COVID-19 and undergoing home isolation were categorized into two groups: those receiving telemonitoring and those receiving standard care without telemonitoring. The primary outcome was all-cause mortality during the home isolation period.

This study was conducted in accordance with the principles of the Declaration of Helsinki and was approved by the Ethics Commission of the University of Milan (Ethics Commission number: 126/20). Written informed consent was obtained from all participants prior to their involvement in the study.

### Participants

The study population comprised residents of Milan, Italy, diagnosed with COVID-19 between October 2020 and February 2021 (during the second wave period). Diagnosis was confirmed by a positive SARS-CoV-2 reverse transcription polymerase chain reaction (RT-PCR) test administered by the Agenzia di Tutela della Salute (ATS) of Milan. Following a positive test, patients were advised to isolate at home for a minimum of 14 days. Resolution of infection was defined as two consecutive negative RT-PCR tests, conducted 48 h apart, after at least 7 days of symptom resolution.

Data were extracted from the ATS health records, including:

• Demographic information (age and sex)
• Presence of pre-existing comorbidities (defined according to ATS records)
• Risk of hospitalization, as determined by an algorithm developed by the ATS during the first wave of the pandemic (categorized as medium or high risk)
• Final outcome (survival or death)

Patients were assigned to the telemonitoring group if they met the ATS criteria for medium or high risk of hospitalization and were within the service’s geographical coverage and capacity. The ATS criteria define high-risk patients as those older than 70 years with specific comorbidities (neurological disorders, chronic heart failure, ischemic heart disease, valvular disease, renal failure, or a recent neoplasm diagnosis) or those who had pneumonia within 15 days of swabbing. Medium-risk patients were those not classified as high risk but with a predicted hospitalization probability of 40% or higher, or if their symptom information was unavailable in the database.^26^ The non-telemonitored group consisted of patients who met the diagnostic criteria but were either outside the telemonitoring service’s coverage area or exceeded its capacity.

### Telemonitoring service (COD19)

The COD19 provided active surveillance for home-isolated COVID-19 patients. This service comprised:

• Remote monitoring of critical clinical parameters.
• Identification of social and health care needs.
• Provision of telemedicine consultations.

The COD19 service operated a call center for 16 h per day, 7 days per week. Resident physicians conducted regular telephone calls to monitor patients’ clinical status throughout their isolation period. Patients exhibiting symptoms or abnormal clinical parameters were promptly referred for infectious disease consultation and potential hospital admission, thereby aiming to reduce inappropriate emergency department visits. The initial contact with patients occurred within 12 h of service activation.^5^

The telemonitoring service was provided free of charge to patients as part of the health care services delivered in accordance with the principles of the Italian National Health Service, which guarantees health care to all citizens regardless of income, employment status, or age.

### Statistical analysis

Continuous variables were presented as median and interquartile range (IQR). Categorical variables were expressed as counts and percentages.

A multivariable logistic regression model was used to assess the association between telemonitoring and mortality (binary outcome: survival/death). The independent variables included:

• Age (continuous, modeled using a linear spline with a node at the median age)
• Sex (categorical: male and female)
• Risk of hospitalization (categorical: medium and high)
• Presence of the eight most frequent comorbidities (binary: yes/no for each)
• Telemonitoring (binary, yes/no)

To assess the model’s goodness-of-fit and to evaluate the optimism of the model’s performance, a bootstrap resampling procedure was performed. Specifically, 1000 bootstrap samples were generated. Using these bootstrap samples, we calculated:

• Somers’ D: to measure the discriminative ability of the model.
• Optimism of R-squared: to assess the overestimation of the model’s explanatory power in the original sample.
• Optimism of the slope: to evaluate the calibration of the model.

This bootstrap procedure allowed us to obtain more robust estimates of the model’s performance and to account for potential overfitting.

A total of 135 deaths were observed in the study cohort. The model had 13 degrees of freedom.

Statistical analyses were performed using R version 4.4.2.

## Results

A total of 6017 patients were included: 2431 (40.4%) in the telemonitoring group and 3586 (59.6%) in the non-telemonitoring group.

Table 1 presents the baseline characteristics of the patients stratified by telemonitoring status. The overall median age was 49 years (IQR: 30, 67). Significant differences were observed between the monitored and non-monitored groups. The monitored group had a significantly higher median age (58 years, IQR: 36, 77) compared with the non-monitored group (44 years, IQR: 27, 58) (*p* < 0.001). The distribution of risk of hospitalization also differed significantly (*p* < 0.001), with a higher proportion of high-risk patients in the monitored group (37%) compared with the non-monitored group (4.7%). Conversely, the non-monitored group had a larger proportion of medium-risk patients (95%) compared with the monitored group (63%). The prevalence of several comorbidities was significantly higher in the monitored group, including cardiovascular disease (43% vs. 19%), endocrine and metabolic disorders (19% vs. 7.8%), renal disease (3.0% vs. 1.3%), neurological disorders (9.7% vs. 3.8%), immunological and rheumatic diseases (13% vs. 7.2%), oncological conditions (12% vs. 5.7%), and digestive system diseases (4.4% vs. 2.0%) (all *p* < 0.001). Skin disease was also slightly more prevalent in the monitored group (0.2% vs. <0.1%, *p* = 0.043). There was no significant difference in sex distribution (*p* = 0.2) between the groups. Notably, the mortality rate was significantly higher in the non-monitored group (2.9%) compared with the monitored group (1.3%) (*p* < 0.001).

**Table 1.** Patients Baseline Characteristics

Characteristic	*N*	Overall *N* = 6,017^a^	Monitored *N* = 2,431^a^	Not monitored *N* = 3,586^a^	*p*-value^b^
Age (years)	6,017	49 (30, 67)	58 (36, 77)	44 (27, 58)	<0.001
Sex	6,017				0.2
Female		2,920 (49%)	1,204 (50%)	1,716 (48%)	
Male		3,097 (51%)	1,227 (50%)	1,870 (52%)	
Risk of hospitalization	6,017				<0.001
Medium		4,947 (82%)	1,531 (63%)	3,416 (95%)	
High		1,070 (18%)	900 (37%)	170 (4.7%)	
Cardiovascular disease	6,017	1,722 (29%)	1,036 (43%)	686 (19%)	<0.001
Endocrine and metabolic disorder	6,017	747 (12%)	467 (19%)	280 (7.8%)	<0.001
Renal disease	6,017	120 (2.0%)	72 (3.0%)	48 (1.3%)	<0.001
Hematological disorder	6,017	8 (0.1%)	3 (0.1%)	5 (0.1%)	>0.9
Neurological disorder	6,017	374 (6.2%)	237 (9.7%)	137 (3.8%)	<0.001
Respiratory disease	6,017	128 (2.1%)	60 (2.5%)	68 (1.9%)	0.13
Immunological and rheumatic disease	6,017	586 (9.7%)	327 (13%)	259 (7.2%)	<0.001
Oncological condition	6,017	503 (8.4%)	300 (12%)	203 (5.7%)	<0.001
Infectious sisease (other than COVID-19)	6,017	45 (0.7%)	17 (0.7%)	28 (0.8%)	0.7
Transplant related condition	6,017	12 (0.2%)	5 (0.2%)	7 (0.2%)	>0.9
Digestive system disease	6,017	181 (3.0%)	108 (4.4%)	73 (2.0%)	<0.001
Associated perinatal and congenital condition	6,017	14 (0.2%)	6 (0.2%)	8 (0.2%)	0.9
Skin disease	6,017	6 (<0.1%)	5 (0.2%)	1 (<0.1%)	0.043
Ill-defined condition	6,017	0 (0%)	0 (0%)	0 (0%)	
Musculoskeletal disease	6,017	2 (<0.1%)	2 (<0.1%)	0 (0%)	0.2
Deceased	6,017	135 (2.2%)	31 (1.3%)	104 (2.9%)	<0.001

^a^Median (Q1, Q3); *n* (%); ^b^Wilcoxon rank sum test; Pearson’s chi-squared test; Fisher’s exact test.

**FIG. 1.**
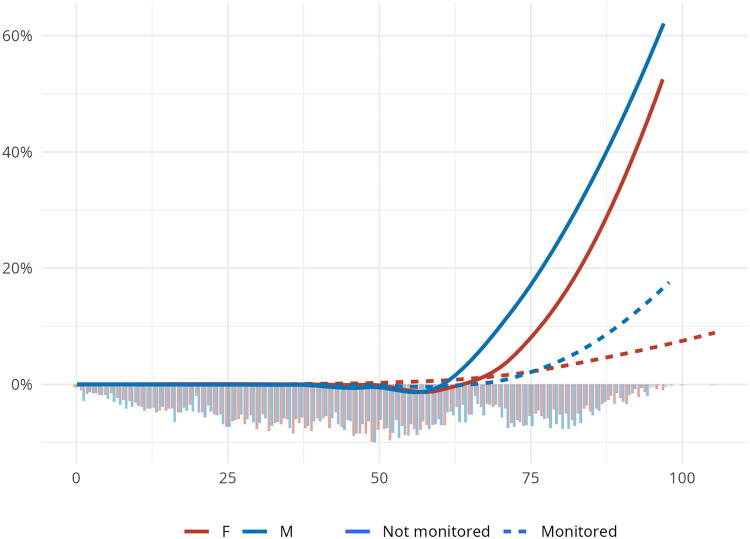
Smooth line (LOESS, Locally Estimated Scatterplot Smoothing) of probability of death of SARS-CoV-2 positive patients during home isolation for each year of age. Superimposed histogram of age distribution by sex.

Figure 1 depicts the relationship between age and the probability of death, stratified by sex and telemonitoring status. The smooth lines (LOESS, Locally Estimated Scatterplot Smoothing) demonstrate a clear age-dependent increase in the probability of death, particularly after the age of 70, in both monitored and non-monitored groups. Notably, the probability of death increases more rapidly with age in the non-monitored group compared with the monitored group. The probability of death appears to be slightly higher in males than in females, especially at older ages.

Table 2 presents the results of the multivariable logistic regression model assessing factors associated with mortality in home-isolated COVID-19 patients. Age significantly influenced mortality, with patients older than 49 years showing a substantially increased odds of death (log[OR] = 5.4, 95% CI: 3.4, 9.6, *p* < 0.001) compared with those younger than 49. Male sex was also associated with significantly higher odds of death (log[OR] = 0.60, 95% CI: 0.17, 1.0, *p* = 0.006). Notably, telemonitoring was associated with a significant reduction in the odds of death (log[OR] = −2.7, 95% CI: −3.2, −2.2, *p* < 0.001). Endocrine and metabolic disorders (log[OR] = 0.48, 95% CI: 0.00, 0.96, *p* = 0.046) and neurological disorders (log[OR] = 0.56, 95% CI: 0.07, 1.0, *p* = 0.023) were also significantly associated with increased mortality. While high risk of hospitalization showed a trend towards increased mortality (log[OR] = 0.46), it did not reach statistical significance (*p* = 0.10).

**Table 2.** Logistic Model of Probability of Death of SARS-CoV-2 Positive Patients during Home Isolation

Characteristic	Log(or)	95% CI	*p*-value
Age (years) <49	0.84	−1.3, 5.2	0.6
Age (years) >49	5.4	3.4, 9.6	<0.001
Sex			
Female	—	—	
Male	0.60	0.17, 1.0	0.006
Risk of hospitalization			
Medium	—	—	
High	0.46	−0.08, 1.0	0.10
Monitored	−2.7	−3.2, −2.2	<0.001
Cardiovascular disease	0.28	−0.31, 0.90	0.4
Endocrine and metabolic disorder	0.48	0.00, 0.96	0.046
Immunological and rheumatic disease	0.15	−0.34, 0.62	0.5
Oncological condition	0.35	−0.13, 0.83	0.15
Neurological disorder	0.56	0.07, 1.0	0.023
Digestive system disease	0.62	−0.02, 1.2	0.052
Respiratory disease	0.10	−1.0, 1.1	0.9
Renal disease	0.46	−0.21, 1.1	0.2

OR, odds ratio; CI, confidence interval.

The logistic regression model’s performance was validated using a bootstrap resampling procedure with 1000 resamples. The model demonstrated good discrimination ability, as indicated by a Somers’ Dxy of 0.8974 after optimism correction. The corrected R-squared value was 0.3366, suggesting a reasonable fit to the data. The slope of the model after correction was 0.614, indicating acceptable calibration. The optimism values for Dxy and R-squared were relatively low, suggesting that the model’s performance was not substantially overestimated in the original sample. These results support the robustness and validity of the logistic regression model in predicting mortality in home-isolated COVID-19 patients.

## Discussion

This study investigated the impact of a telemonitoring service (COD19) on the mortality rate of COVID-19 patients undergoing home isolation in Milan, Italy, during the second wave of the pandemic. Our findings revealed that telemonitoring was associated with a significant reduction in mortality. Patients receiving telemonitoring had a mortality rate of 1.3%, compared to 2.9% in those receiving standard care without telemonitoring. Several factors were associated with increased mortality in this population. Older age was a strong predictor of death, with patients older than 49 years having substantially higher odds of death compared with younger patients. Male sex was also associated with a higher likelihood of death. In addition, certain comorbidities, such as endocrine and metabolic disorders and neurological disorders, increased the risk of mortality.

The observed reduction in mortality among telemonitored patients is likely due to several factors. Telemonitoring enabled continuous monitoring of patients’ clinical conditions, allowing for early identification of deterioration and prompt intervention. This proactive approach helped prevent severe complications and reduced the need for emergency hospitalizations. In addition, telemonitoring facilitated communication between patients and health care providers, ensuring that patients received timely and appropriate care. These results are in line with results reported by Thompson et al.,^16^ who found a significant association between remote monitoring and reduced mortality, reporting an adjusted odds ratio of 0.50 for death in the monitored group. Our study similarly observed a lower mortality rate in the telemonitored group. Moreover, they also found a nonsignificant decrease in other health care utilization outcomes, suggesting that remote monitoring may have broader benefits beyond mortality reduction. Sherlaw-Johnson et al.^8^ did not find a statistically significant impact of the CO@h program on mortality. The author^8^ suggests that this lack of significance may be due to several factors, including low enrollment rates, incomplete data, and variations in implementation across different sites. The Haddad et al.^27^ study, focused on high-risk patients, also found that RPM was associated with a significant reduction in mortality. In their study, the mortality rate for RPM-engaged patients was 0.5%, compared to 1.7% for non-engaged patients. This finding further supports the conclusion that remote monitoring can play a crucial role in reducing mortality among COVID-19 patients.

As highlighted in our report, telemonitoring applications play a crucial role in maintaining continuous patient oversight between clinic visits. They facilitate early detection of health deterioration, enabling timely interventions and ultimately leading to improved patient outcomes.^6^ In addition, remote monitoring minimizes unnecessary clinic visits and boosts health care provider efficiency by delivering real-time patient data.^14^ RPM technology grants clinicians access to essential symptom and condition details that patients may not disclose during in-person consultations. It ensures a more accurate evaluation of a patient’s health, contributing to more effective treatment decisions.^28^ Furthermore, these technologies strengthen communication between patients and health care providers, supporting timely interventions through alerts and proactive follow-ups. Finally, the storage of patient data supports visualization of long-term health trends and helps determine ideal treatment plans.^29^

Future research in telemedicine will increasingly focus on evaluating the effectiveness of telemonitoring across diverse patient populations and health care settings. A key aspect will be assessing the adaptability of telemedicine to different patient groups, including those with chronic conditions, disabilities, elderly individuals, and patients with limited access to health care. Understanding how these technologies can be tailored to meet specific patient needs is essential for improving their effectiveness. In addition, analyzing telemonitoring applications across various health care settings, such as rural versus urban environments and low-income countries with limited health care access, will provide valuable insights into its integration within existing health care systems.^30,31^

Telemedicine has become a transformative element in health care, especially in enhancing access to medical services.^31,32^ Ongoing telemedicine research will play a crucial role in optimizing telemonitoring, ensuring its seamless integration into global health care systems, expanding health care accessibility, and ultimately enhancing patient outcomes.^32–34^ The COVID-19 pandemic’s unprecedented strain on health and social services exposed vulnerabilities and a lack of preparedness in modern health systems worldwide.^35^ Based on evidence from the pandemic, telemedicine may serve as a valuable tool for emergency health care management in natural disasters, pandemics, or mass casualty events.^35–38^

This study has several strengths, including its large sample size and its focus on a real-world population of COVID-19 patients. However, it also has some limitations, including its retrospective design, which cannot definitively establish causality, and the potential for residual confounding due to unmeasured factors. Patients were not randomly assigned to the telemonitoring and non-telemonitoring groups, which could have led to systematic differences between the groups that influenced the outcome independently of the intervention. Nonetheless, despite the telemonitored group being older and having a higher prevalence of comorbidities, factors typically associated with increased mortality risk, this group demonstrated a significantly lower mortality rate compared with the non-telemonitored group. For this reason, we think the study provides strong evidence that telemonitoring is associated with a significant reduction in mortality among COVID-19 patients undergoing home isolation.

## Conclusion

Our findings indicate that a telemedicine model incorporating proactive at-home monitoring is linked to reduced mortality rates in COVID-19 patients. Remote monitoring has proven effective for the early detection of health decline, enabling timely interventions that ultimately enhance clinical outcomes. This approach promotes more efficient disease management and reduces hospitalization rates, offering new strategic opportunities for managing severe health conditions. Future research will be vital in evaluating the effectiveness of telemonitoring across diverse patient populations and health care settings, which is essential for the successful integration of telemedicine into health care systems. Considering the lessons learned from the COVID-19 crisis, telemonitoring could be a key strategy for crisis preparedness in emergency health care management.

## Abbreviations Used

ATS: Agenzia di Tutela della Salute
COD: Operations center for discharged patients
IQR: Interquartile range
LOESS: Locally estimated scatterplot smoothing
RPM: Remote patient monitoring
RT-PCR: Reverse transcription polymerase chain reaction
